# The Central Role of Multiple P450 Genes and Their Co-factor CPR in the Development of Permethrin Resistance in the Mosquito *Culex quinquefasciatus*

**DOI:** 10.3389/fphys.2021.802584

**Published:** 2022-01-13

**Authors:** Youhui Gong, Ting Li, Qi Li, Shikai Liu, Nannan Liu

**Affiliations:** ^1^Department of Entomology and Plant Pathology, Auburn University, Auburn, AL, United States; ^2^State Key Laboratory for Biology of Plant Diseases and Insect Pests, Institute of Plant Protection, Chinese Academy of Agricultural Sciences, Beijing, China; ^3^Department of Biological Sciences, Alabama State University, Montgomery, AL, United States; ^4^College of Aquaculture, Ocean University of China, Qingdao, China

**Keywords:** P450 genes, permethrin resistance, metabolite resistance, *in vitro* metabolism, cytochrome P450-reductase, co-overexpression

## Abstract

Mosquitoes’ increasing resistance to insecticides is becoming a major threat for control efforts worldwide. Multiple P450 genes that are up-regulated in permethrin resistant strains of *Culex quinquefasciatus* have been linked to the development of resistance. In the current study, we characterized the function of six P450 genes, CYP6P14, CYP6BZ2, CYP9J33, CYP9J34, CYP9J40, and CYP9J45, that are overexpressed in the permethrin resistant *Culex* mosquitoes and showed their capability in metabolism of permethrin. These six P450 genes can convert 3-phenoxybenzoic alcohol (PBCHO) to a less toxic product, 3-phenoxybenzoic acid (PBCOOH), indicating that these P450s play an important role in permethrin degradation pathways. Although we know multiple P450 genes are over-expressed in permethrin resistant *Culex* mosquitoes, it remains to be seen whether cytochrome P450-reductase (CPR) gene that are co-overexpressed with P450 genes in permethrin resistant mosquitoes do indeed serve as a resistance mechanism. An in-depth investigation of the expression of CPR gene in resistant mosquitoes was conducted in permethrin resistant mosquitoes. The finding of CPR gene overexpression in permethrin resistant mosquitoes suggested the importance of co-overexpression of multiple P450 genes with their obligatory electron donor CPR in the complex detoxification system, boosting the metabolism of permethrin and hence the development of permethrin resistance in *Cx. quinquefasciatus*.

## Introduction

The mosquito *Culex quinquefasciatus* Say is a primary vector of lymphatic filariasis, St. Louis encephalitis and West Nile encephalitis, making it an important target for control efforts in many countries (Maizels and Denham, 1992; Harish Kumar et al., 2011). Preventing the spread of mosquito-borne diseases relies heavily on the use of insecticides and pyrethroids are by far the most important class of insecticides currently used for the control of mosquito vectors, being used for insecticide treated bednets and indoor residual spraying (van den Berg et al., 2012). However, the development of insecticide resistance in *Culex* mosquitoes is rapidly becoming a major problem for mosquito control efforts worldwide (Liu, 2008, 2015; Fofana et al., 2012).

Cytochrome P450s are critical for the detoxification and/or activation of xenobiotics and endogenous compounds (Feyereisen, 2005), generally through oxidation in the presence of either their obligatory electron donor NADPH cytochrome P450-reductase (CPR) or cytochrome b5 (Cyt b5) (Murataliev et al., 2008). There is a growing body of evidence to suggest that P450 genes are involved in the development of resistance to pyrethroid insecticides in mosquitoes (Müller et al., 2008; Komagata et al., 2010; Stevenson et al., 2012; Riveron et al., 2013; Smith et al., 2019; Vontas et al., 2020). Multiple P450 genes are known to be up-regulated in permethrin resistant mosquitoes (Yang and Liu, 2011; Reid et al., 2012; Yang et al., 2021). The knockdown of several overexpressed P450 genes in resistant *Culex* mosquitoes using RNA interference (RNAi) has confirmed their involvement in insecticide resistance (Li et al., 2015; Yang et al., 2021). A study utilizing TALENs and the CRISPR technique has further demonstrated the role of CYP9M10 in conferring permethrin resistance in *Culex* mosquitoes (Itokawa et al., 2016). A functional study utilizing heterogenous expression and *in vitro* metabolism study has shown that two genes, CYP9M10 and CYP6AA7, can not only metabolize permethrin, but also metabolize the two main permethrin metabolites, PBOH and PBCHO (Gong et al., 2017), further revealing their important role in permethrin degradation pathways. However, as yet there have been no reports regarding the permethrin metabolism function of other P450 genes that are also known to be over-expressed in permethrin resistant *Culex* mosquitoes.

Cytochrome P450 reductase (CPR) is an essential electron transfer protein located on the endoplasmic reticulum of most cell types that plays an important role in cytochrome P450-mediated drug and insecticide metabolism (Yamazaki et al., 2002; Kashiwada et al., 2005; Murataliev et al., 2008; Traylor et al., 2017). Because CPR can act as obligatory electron donors for P450s, it may also be linked to the development of insecticide resistance in insects (Liu and Scott, 1996; Zhang and Scott, 1996). In some pest insects, it is not only P450 genes that are up-regulated in insecticide resistance strains; CPR is also co-over-expressed (Nikou et al., 2003; Marcombe et al., 2012; Edi et al., 2014; Chen and Zhang, 2015), suggesting its possible role in insecticide resistance through co-expression with functional P450s. Moreover, CPR has also been linked to P450 mediated detoxification and insecticide resistance by *in vivo* RNAi analysis in some insects (Lycett et al., 2006; Zhu et al., 2012; Liu et al., 2015; Zhang et al., 2015, 2017). Although we know multiple P450 genes are over-expressed in permethrin resistant *Culex* mosquitoes, it remains to be seen whether CPR gene that is co-over-expressed with P450 genes in permethrin resistant mosquitoes do indeed serve as a resistance mechanism. An in-depth investigation of the expression of CPR gene in resistant mosquitoes should thus confirm its role, if any, in cytochrome P450-mediated insecticide metabolism and hence its possible link to permethrin resistance.

In the current study, we heterogeneously expressed six P450 proteins with Culex mosquito CPR in baculovirus expression system. These P450 genes belong to the CYP6 and CYP9 families and have showed to be overexpressed in either one of two permethrin resistant strains (CYP9J40 in HAmCq^G8^ or CYP9J33 in MAmCq^G6^) or both of them (CYP9J34, CYP6P14, CYP9J45, and CYP6BZ2 are both in HAmCq^G8^ and MAmCq^G6^). Although the association between changes in the expression levels of some of these P450 genes and the levels of insecticide resistance in mosquitoes have been confirmed using RNAi analyses (Yang et al., 2021), *in vitro* metabolism study still further need to demonstrated their precise role in the metabolism pathways of permethrin. In this study, their co-factor, CPR, was also investigated in gene expression in resistant strain to further confirm its role in these P450 mediated detoxification system.

## Materials and Methods

### Mosquito Strains

Three mosquito strains of *Cx. quinquefasciatus* were used in this study. HAmCq^G8^ is the eighth generation of permethrin-selected offspring of the HAmCq^G0^ strain, which was collected from Huntsville County, AL (Li and Liu, 2010); MAmCq^G6^ was the sixth generation of permethrin-selected offspring of the MAmCq^G0^ strain, which was collected from Mobile County, AL (Li and Liu, 2010); S-Lab is an insecticide susceptible strain kindly provided by Dr. Laura Harrington (Cornell University, Ithaca, NY, United States). HAmCq^G8^ and MAmCq^G6^ were highly resistant to pyrethroid insecticides after permethrin selections and exhibit a 2,700- and 570-fold levels, respectively, of resistance to permethrin at the fourth-instar larval stage and 100- and 15-fold at the adult stage compared to susceptible S-lab (Xu et al., 2006; Li and Liu, 2010). The HAmCq^G8^ and MAmCq^G6^ were maintained in the laboratory and was pressured with permethrin biannually. In the time for the study by Gong et al. (2017), the resistance level of HAmCq^G8^ strain was 2,714-fold compared to S-Lab strain.

In the current study, six P450 genes were chosen for *in vitro* protein expression and metabolism study. majority of which (CYP9J34, CYP6P14, CYP9J45, and CYP6BZ2) were overexpressed in both HAmCq^G8^ and HAMCq^G6^ stains except CYP9J40 that was uniquely overexpressed in HAmCq^G8^ and CYP9J33 that was uniquely overexpressed in HAMCq^G6^. All the mosquitoes were reared at 25 ± 2°C under a photoperiod of 12: 12 (L: D) h (insectary conditions) and fed blood samples from horses (Large Animal Teaching Hospital, College of Veterinary Medicine, Auburn University, Auburn, AL, United States).

### Construction of pENTR™ Expression Plasmids Containing CYP Genes

CYP9J33 gene was cloned from MAmCq^G6^ strain mosquitoes because this gene was uniquely overexpressed in MAmCq^G6^ resistant strain; all other CYP genes (CYP9J34, CYP6P14, CYP9J45, CYP6BZ2, and CYP9J40) were cloned from HAmCq^G8^ strain mosquitoes. The methods used for the total RNA extraction and cDNA synthesis are as described by Gong et al. (2013). The gene-specific primers used for cloning the complete reading frames of the CYP genes were designed according to the *Cx. quinquefasciatus* genome sequence^1^ (Table 1). Construction of pENTR™ expression plasmids containing CYP genes were according the methods described in Gong et al. (2017). pENTR™ plasmids with CYP genes were purified using the PureLink HQ Mini plasmid purification Kit (Invitrogen, Carlsbad, CA, United States) following the manufacturer’s instructions. Orientation of the inserted CYP genes was tested by PCR using the forward primer of each specific gene and the reverse primer of M13, as per the manufacturer’s instructions (Invitrogen). Expression plasmids were further sequenced for validation. pENTR™ plasmids with the CPR gene were obtained from the study of Gong et al. (2017).

**TABLE 1:** Primers of genes used for ORF full length cloning (CYP genes) or qPCR analysis (CPR).

Transcript ID	Accession no.	Gene	Primer	
			Forward	Reverse
CPIJ005955	XM_001847351.1	CYP6P14	CACCATGGCACTTTTGAGTCTAATGT	TCAAATCTTATCAACCCTAAGGTG
CPIJ005956	XM_001847352.1	CYP6BZ2	CACCATGGACGGACTGCAGAGTATCT	CTAAATCCGTTCCACCAGCAGC
CPIJ010537	XM_001855163.1	CYP9J45	CACCATGGTCTCCGTAGACCTGTT	TCACATTCTTGGTTTAAACTCC
CPIJ010543	XM_001855188.1	CYP9J40	CACCATGGAGGTGAACATCGTAT	TCACTTCCTTAGTTTCAGTCGC
CPIJ010544	XM_001855191.1	CYP9J33	CACCATGGAAGTAGATCTATTGTACGT	TTAAACTTTTCGTGGTTTAAAGT
CPIJ010546	XM_001855208.1	CYP9J34	CACCATGCAGATAGATCTTGCGTAC	TCATTTTCTTGGTCTAAACTCC
CPIJ014664	XM_001865766.1	CPR	GCTTTATCCAGGAGCGAGATT	CTTGACGTAGTCTTCGAGTTCC
	AY_988447	18S RNA	CGCGGTAATTCCAGCTCCACTA	GCATCAAGCGCCACCATATAGG

### Recombinant Baculovirus Expression of CYP and CPR in Sf9 Cells

Recombinant baculovirus containing CYP and CPR was constructed by incubating pENTR™ expression plasmids of CYP or CPR with BaculoDirect Linear DNA and LR clonase™ II enzyme mix overnight at 25°C as per the BaculoDirect™ Baculovirus Expression system manufacturer’s instructions (Invitrogen). The recombinant baculovirus containing CYP or CPR was transfected into *Spodoptera frugiperda* (Sf9) cells using CellfectinR II Reagent (Invitrogen) to produce a series of recombinant baculovirus stock solutions. The preparation of large-scale high titer stocks of recombinant baculovirus for the expression of proteins in insect cells was performed according to the manufacturer’s instructions (Invitrogen). The baculovirus titer was measured by a plaque forming assay and a titer of ∼2 × 10^8^ pfu/mL was used as the final stock for infection of the Sf9 cell in the large-scale amplification of the co-expressed CYP/CPR proteins (Gong et al., 2017). To co-express P450 and CPR (CYP/CPR) in Sf9 cells, the final stocks of P450- and CPR- recombinant baculovirus were co-transfected in Sf9 cells and 1 μg/mL hemin and 0.1 mM 5-ALA (Gong et al., 2017) were added to the culture medium 24 h after Sf9 cell co-infection. A multiplicity of infection (MOI) ratio of 10:1 (0.5:0.05) for the co-expression of the CYP/CPR-recombinant baculovirus in Sf9 cells was used (Gong et al., 2017).

### P450 Content, CYP and CPR Activity Determination

The preparation of the microsomal proteins was as described by Gong et al. (2017). To assay the P450 content, microsomal protein was measured and analyzed using a UV/visible spectrophotometer (DU640, Beckman Coulter, United States) according to the procedure described by Liu and Scott (1996). P450-mediated activity was estimated by measuring 7-ethoxycoumarin *O*-deethylase (ECOD assay) activity following the procedure described in Gong et al. (2017). CPR activity was determined using the cytochrome c assay described by Gong et al. (2017).

### HPLC Analysis and Permethrin, PBOH and PBCHO Metabolism Study

The method and procedure used for the metabolism experiments were as described in our previous studies (Gong et al., 2017; Feng and Liu, 2020) with minor modifications. The *in vitro* reactions contained 20 μM permethrin and either 20 μM PBOH or 10 μM PBCHO, 1 mg of CYP/CPR microsomes, 0.25 mM MgC_*l*2_, and 1 mM NADPH for a total reaction volume of 700 μL; NADPH was omitted in the control reaction. After the incubation at 30°C for 2 h, the reaction was stopped by adding 700 μL of acetonitrile and incubated with shaking for an additional 20 min. After that, the mixture was centrifuged at 16,000 rmp for 10 min and the supernatant was collected by filtering through 0.45 μm membranes and transferred to ultraclean glass vials for HPLC analysis. The injection volume was 10 μL. Reactions were performed in triplicate and a paired *T*-test of sample reactions (with NADPH) vs. the control (without NADPH) was performed for the statistical measurements of substrate depletion following the method described by Stevenson et al. (2012).

Insecticide metabolism was monitored by reverse-phase HPLC on an HPLC system (Alliance Waters 2695) equipped with a Nova-Pak C18 column (60Å, 4 μm, 3.9 mm × 15 0mm, 1/pkg [WAT086344]) and a Waters 2487 Dual λ absorbance detector. Two solvents (solvent A: 90% acetonitrile and 10% H_2_O, solvent B: 95% water, 5% acetonitrile adjusted to pH 2.3 with 85% phosphoric acid) were used for the gradient elution (flow rate: 1 mL/min). The gradient system (linear increase) was initiated with 50% of solvent A and 50% of solvent B, reaching 75% of solvent A at 6 min and then 100% of solvent A at 8 min. It was maintained at this level for 4 min and then reduced to 50% at 13 min, where it was kept for a further 4 min to return the column to the initial conditions (method according to Choi et al., 2002, with some modifications). The chromatographic analysis was conducted at 23°C and monitored by tracking the absorbance at 232 nm. Permethrin and metabolites PBOH, PBCHO, and PBCOOH were identified by comparing retention times with analytical standards (Choi et al., 2002). Permethrin concentration was measured as the total area under the two peaks of *trans*-permethrin and *cis*-permethrin (Stevenson et al., 2012), and was quantified by peak integration (Chromeleon software, Dionex, Thermo Fisher Scientific, Waltham, MA, United States) and calculated based on a standard curve for permethrin (*y* = 16670*x* + 5068.5, *R*^2^ = 0.9996) conducted in this study. The metabolic rates of permethrin for the 2 h reactions were also calculated and the results expressed as pmol substrate/min/pmol P450.

### Expression Profiles of CPR in Development Stages of Different Strains

The expression profiles for CPR gene in larvae, females and males of two mosquito strains (HAmCq^G8^ and S-Lab), one highly resistant and the other susceptible to permethrin, were analyzed. Total RNA was isolated from HAmCq^G8^ and S-Lab strains at different life stages using the acidic guanidine thiocyanate phenol– chloroform method (Liu and Scott, 1997). The DNA was removed from the total RNA (5 μg) of each mosquito sample using DNase (TURBO DNA-free, Ambion, Austin, TX, United States) and the DNA-free total RNA (0.5 μg per sample) was then reverse-transcribed to cDNA using a Transcriptor First Strand cDNA Synthesis kit (Roche Applied Science, Mannheim, Germany) and a random hexamer primer following the manufacturer’s instructions. The DNA-free RNA content was quantified at an absorbance of 260 nm, and the RNA quality assessed at OD260/280 in a spectrophotometer. Real time PCR was performed with the SYBR Green master mix Kit and ABI 7500 Real Time PCR system (Applied Biosystems). Each qPCR reaction (25 μL final volume) contained 1× SYBR Green master mix, 1 μL of cDNA (400 ng), and the gene specific primer pair (Table 1) at a final concentration of 3–5 μM. The primers of CPR for qPCR analysis were designed according to the *Cx. quinquefasciatus* genome sequence (see Text Footnote 1), and the fragments (128 bp) of this gene were cloned and vindicated by DNA gel electrophoresis and sequencing. All samples, including the ‘no-template’ negative control, were performed in triplicate. The reaction cycle consisted of a melting step of 50°C for 2 min, then 95°C for 10 min, followed by 40 cycles of 95°C for 15 s and 60°C for 1 min. Specificity of the PCR reactions (for checking non-specific amplification) was assessed by a melting curve analysis for each PCR reaction using Dissociation Curves software (Wittwer et al., 1997). Relative expression levels for CPR gene were calculated by the 2^–ΔΔ*CT*^ method using SDS RQ software (Livak and Schmittgen, 2001). The 18S ribosome RNA gene (GenBank: AY988447.1), as an endogenous control was used to normalize the expression of target genes according to our previous study that the 18S ribosome RNA gene expression remained constant among all mosquito strains, including S-Lab and HAmCq^G8^ (Liu et al., 2011; Yang and Liu, 2011; Reid et al., 2012; Gong et al., 2021). With the primer designed for 18S ribosome RNA (Table 1), the 159 bp PCR amplicon was generated in all mosquito samples. Each experiment was repeated three times with different preparations of RNA samples.

### Induction Expression of CPR Following Permethrin Treatment

Induction of the CPR gene in response to permethrin treatment with time was determined by treating ∼1,000 late 3rd instar larvae of each of two *Culex* mosquito strains (HAmCq^G8^ and S-Lab) with permethrin at their respective LC_50_ concentrations (0.005 and 10 ppm for the S-Lab and HAmCq^G8^ strains, respectively) (Gong et al., 2013). The insecticide treatment method was performed as our previous description (Liu et al., 2004; Gong et al., 2017). Briefly, the permethrin concentrations of LC50s (0.005 ppm for Slab) and (10 ppm for HAmCq^G8^) were prepared using tap water with 1% acetonitrile. The survivors were collected after 12, 24, 48, and 72 h treatment and stored in a −80°C ultralow temperature freezer for subsequent RNA extraction and cDNA synthesis (Gong et al., 2013). The relative mRNA expression of CPR gene was examined between LC_50_ concentration treatments and control treatments in each strain (S-Lab and HAmCq^G8^ strains, respectively). qRT-PCR was performed as above. Control mosquitoes exposed to 1% acetone were collected at the same time points as their permethrin treatment counterparts. The experiments were repeated three times.

### Gene Expression Significance Analysis

For overexpression of CPR in resistant strain as compared to S-Lab strain and induction expression of CPR in response to permethrin treatment, a student *T*-test was used for the statistical significance analysis, only both a value of *P* ≤ 0.05 and a cut-off value of a ≥2-fold change in expression (Strode et al., 2008) was considered as a statistically significant overexpression (or induction).

## Results

### P450 Content, CPR Activity, and P450 Activity

Each of the P450s was co-expressed with CPR by simultaneous infection of Sf9 cells through two recombinant viruses, P450-recombinant baculovirus (P450rbv) with MOI 0.5 and CPR-recombinant baculovirus (CPRrbv) with MOI 0.05, in the suspension culture (Gong et al., 2017). Microsomal proteins from the co-expressed P450/CPR in the Sf9 cells were isolated 72 h post-infection and used for the biochemical characterization study. The parent sf9 cells showed no-detectable P450 content, however, reduced CO-P450/CPR exhibited a characteristic maximum absorption peak at 450 nm with P450 contents of 145.9, 104.7, 123.6, 449.5, 154.9, and 128.6 pmol/mg protein for CYP6P14/CPR, CYP6BZ2/CPR, CYP9J40/CPR, CYP9J45/CPR, CYP9J33/CPR, and CYP9J34/CPR, respectively, showing that all these CYPs were successfully expressed in the Sf9 cells (Table 2). The parent sf9 cells exhibited cytochrome c activity (CPR activity) with 21.2 nmol/min/mg protein. The CPR activity of 43.6–162.5 nmol/min/mg protein for the co-expressed CYP/CPR further confirmed the successful co-expression of CPR with CYP in the insect cells (Table 2).

**TABLE 2:** The P450 content and CPR activity of the co-expressed CYP/CPR microsomes.

CYP isomers	P450 content pmol/mg	CPR activity nmol/min/mg
Sf9 cells	/	21.2 ± 1.2
CYP6P14/CPR	145.9 ± 2.1	125.0 ± 2.6
CYP6BZ2/CPR	104.7 ± 5.1	84.9 ± 2.0
CYP9J40/CPR	123.6 ± 3.2	81.3 ± 1.7
CYP9J45/CPR	449.5 ± 13.6	162.5 ± 4.3
CYP9J33/CPR	154.9 ± 6.1	90.5 ± 2.1
CYP9J34/CPR	128.6 ± 4.6	43.6 ± 1.1

*“/” represents “not detected.”*

The ECOD activities of all the CYP/CPR proteins and protein isolated from the sf9 cells were tested (Table 3). No significant P450 activity was observed in the proteins isolated from the original Sf9 cells. CYP6P14/CPR had an even lower ECOD activity than the parental sf9 cells with no CYP/CPR expression, revealing that CYP6P14/CPR cannot convert significant amounts of the substrate 7-ethoxycoumarin to the fluorescent product 7-hydroxycoumarin. The other CYP/CPRs all metabolized 7-ethoxycoumarin with different efficiencies, with turnover rates (*K*cat) of 1.22, 0.70, 0.34, 3.56, and 10.86 pmol/min/pmol P450 for CYP6BZ2/CPR, CYP9J40/CPR, CYP9J45/CPR, CYP9J33/CPR, and CYP9J34/CPR, respectively.

**TABLE 3:** ECOD activity of co-expressed CYP/CPR microsomes.

CYP isomers	ECOD activity	
	pmol/min/mg	pmol/min/pmol P450
Sf9 cells	28.8 ± 2.5	/
CYP6P14/CPR	15.7 ± 1.0	0.11 ± 0.05
CYP6BZ2/CPR	127.9 ± 5.4	1.22 ± 0.20
CYP9J40/CPR	86.6 ± 2.8	0.70 ± 0.15
CYP9J45/CPR	152.7 ± 7.6	0.34 ± 0.12
CYP9J33/CPR	1682.4 ± 12.3	10.86 ± 2.10
CYP9J34/CPR	458.4 ± 9.5	3.56 ± 0.56

*“/” represents “not detected.”*

### Permethrin Metabolism

**FIGURE 1:**
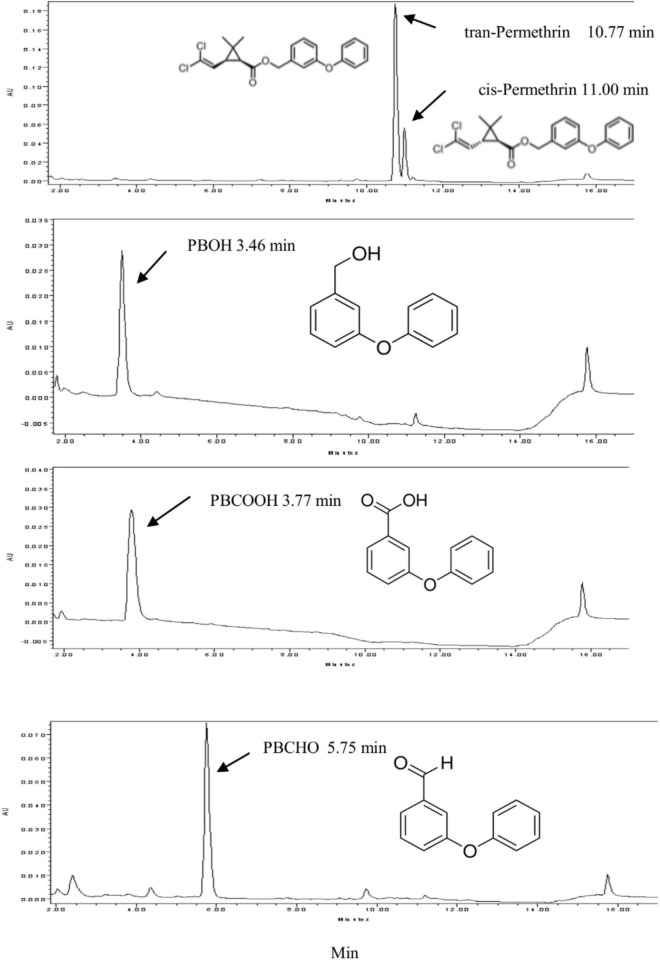
The elution times of permethrin, PBOH (3-phenoxybenzoic alcohol), PBCHO (3-phenoxybenzaldehyde) and PBCOOH (3-phenoxybenzoic acid) in the HPLC analysis. The elution products were monitored via their absorption at 232 nm, with elution times for *trans*- and *cis*- permethrin of 10.77 and 11.00 min, respectively, for PBOH of 3.46 min, and for PBCHO and PBCOOH of 5.75 and 3.77 min, respectively. 16 μM PBOH, PBCHO, and PBCOOH and 32 μM permethrin were used in this standard curve, respectively.

**FIGURE 2:**
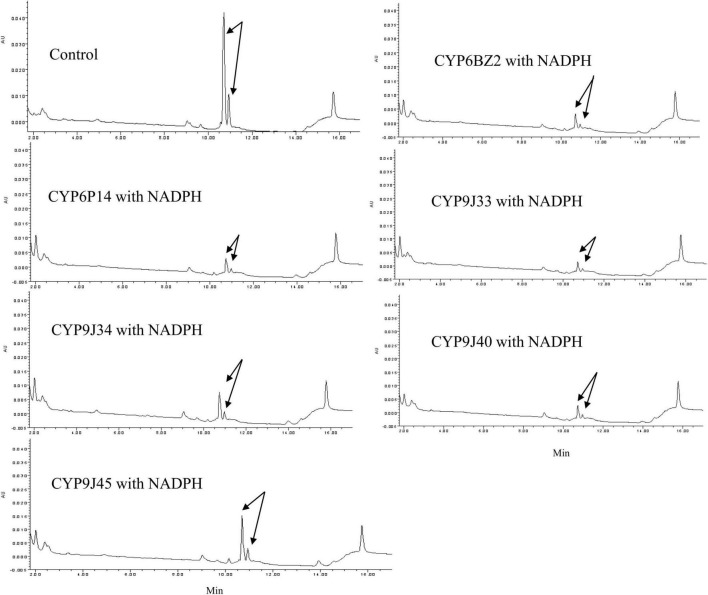
HPLC profiles of permethrin metabolism mediated by expressed CYP/CPR isomers. Black arrows indicate the substrate permethrin. Reactions were performed with 20 μM permethrin, 1 mM NADPH, 0.25 mM MgCl_2_ and 1 mg expressed microsome CYP/CPR proteins incubated for 2 h at 30°C. Each CYP/CPR protein with NADPH has a control reaction with the same microsome without NADPH. However, only the chromatogram of control reaction CYP6BZ2 without NADPH was shown in this figure.

Permethrin metabolism was assayed with microsomal proteins of CYP/CPR in the presence or absence of NADPH. The degradation of the substrate was monitored by reverse-phase HPLC. For the HPLC analysis, the *trans*-/*cis*-permethrin elution times were 10.77 and 11.00 min, respectively (Figure 1). Three permethrin common intermediate metabolites, PBOH, PBCHO, and PBCOOH, were tested, with elution times of 3.46, 5.75, and 3.77 min, respectively (Figure 1). The permethrin (mixture of *cis*-and *trans*- isomers) was significantly metabolized by CYP9J33/CPR, CYP9J34/CPR, CYP9J40/CPR, CYP9J45/CPR, CYP6BZ2/CPR, and CYP6P14/CPR with decreases of 91.5, 76.3, 87.5, 60.7, 87.0, and 87.3%, respectively, in the total 20 μM permethrin compared to the no-NADPH control after a 120 min incubation period (Figure 2), clearly demonstrating that all are capable of metabolizing permethrin *in vitro*. CYP9J33/CPR, CYP9J34/CPR, CYP9J40, CYP9J45, CYP6BZ2, and CYP6P14 metabolized the permethrin with metabolite rates of 0.34 ± 0.10, 0.35 ± 0.05, 0.41 ± 0.10, 0.08 ± 0.00, 0.49 ± 0.06, and 0.35 ± 0.05 pmol/min/pmol P450, respectively, for permethrin clearance (Table 4). Based on the chromatographic analysis, no metabolites of PBOH were eluted (Figure 2), indicating either that permethrin was converted into PBOH at undetectable levels, or the presence of other metabolites that were not detected under these HPLC conditions.

**TABLE 4:** Permethrin metabolization rates of expressed CYP/CPR microsomes.

Metabolite rate (pmol/min/pmol P450)					
CYP9J33	CYP9J34	CYP9J40	CYP9J45	CYP6BZ2	CYP6P14
0.34 ± 0.1	0.35 ± 0.05	0.41 ± 0.1	0.08 ± 0.02	0.49 ± 0.06	0.35 ± 0.05

### Metabolites PBOH and PBCHO Metabolism

**FIGURE 3:**
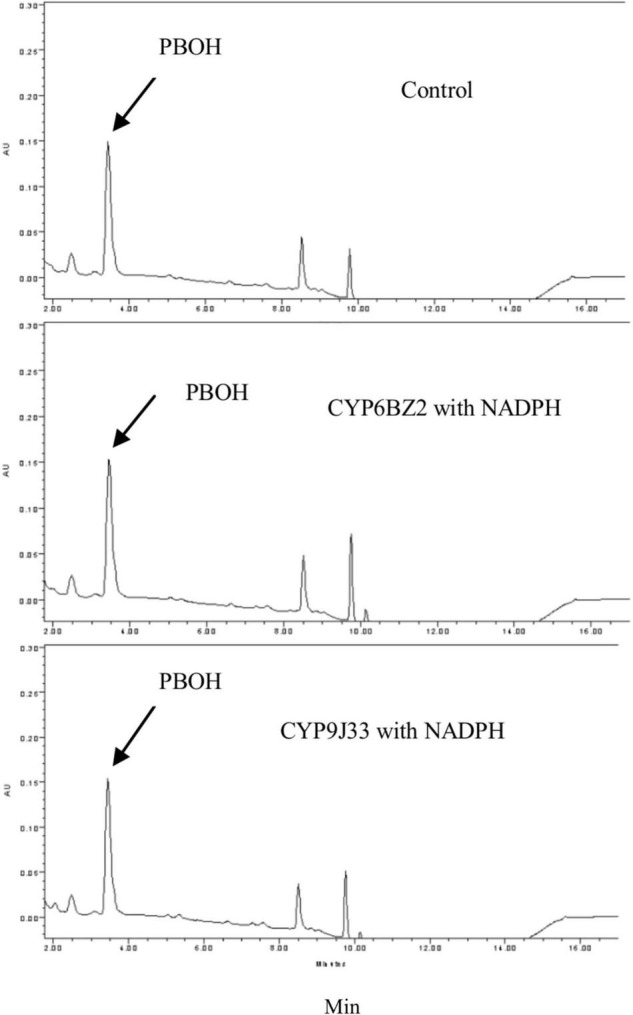
HPLC profiles of PBOH (3-phenoxybenzoic alcohol) metabolism mediated by expressed CYP/CPR microsomes (CYP6BZ2 and CYP9J33 shown as an example). Black arrows indicate the substrate PBOH. The *in vitro* reactions contained 20 μM PBOH, 1 mg of CYP/CPR microsomes, 0.25 mM MgCl_2_, and 1 mM NADPH for a total reaction volume of 700 μL; NADPH was omitted in the Control reaction. Each CYP/CPR protein with NADPH has a control reaction with the same microsome without NADPH. However, only the chromatogram of control reaction CYP6BZ2 without NADPH was shown in this figure.

When PBOH was used as a substrate (elution time descried in Figure 1), in the presence of NADPH no decrease in the level of PBOH (measured in terms of peak area) was detected for reactions with any of the CYP/CPRs compared to controls with no NADPH present. No metabolite PBCOOH was detected in any of the sample based on a comparison with the chromatographic profile obtained for a PBCOOH standard (the curves for CYP9J33 and CYP6BZ2 are shown in Figure 3 as examples).

**FIGURE 4:**
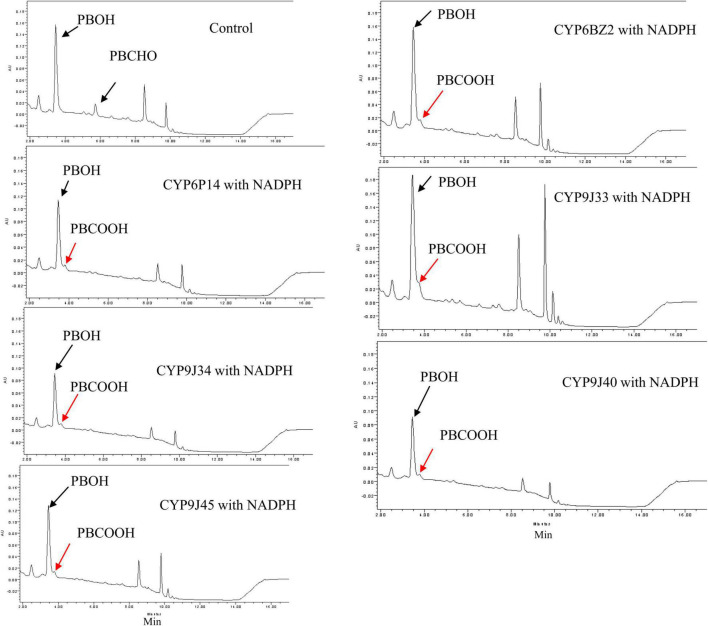
HPLC profiles of PBCHO (3-phenoxybenzaldehyde) metabolism mediated by expressed CYP/CPR microsomal proteins. Black arrows indicate the substrate PBCHO, or the reduced conversion: PBOH. Red arrows indicate the oxidation product PBCOOH. The *in vitro* reactions contained 10 μM PBCHO, 1 mg of CYP/CPR microsomes, 0.25 mM MgCl_2_, and 1 mM NADPH for a total reaction volume of 700 μL; NADPH was omitted in the Control reaction. Each CYP/CPR protein with NADPH has a control reaction with the same microsome without NADPH. However, only the chromatogram of control reaction CYP6BZ2 without NADPH was shown in this figure.

When PBCHO was used as a substrate (elution time descried in Figure 1), the conversion of PBCHO to PBCOOH increased slightly when any of the CYP/CRP reactions with the present of NADPH, but not in the reactions without NADPH (the control, Figure 4), confirming that all the CYPs were capable of metabolizing PBCHO into PBCOOH as long as NADPH was also present, albeit with lower activities. Based on the chromatographic analysis, PBCHO decreased considerably in all the sample reactions, but, however, much of the PBCHO was reduced into PBOH, only very low levels of PBCOOH from the oxidization reaction were detected, suggesting that there was no equilibrium between the oxidation and reduction reactions. This result disagrees with Chandor-Proust et al. (2013), who found an equilibrium between oxidation and reduction reactions for PBCHO.

### Overexpression and Induction of CPR in Highly Resistant HAmCq^G8^ Strain Compared to S-Lab Strain

**FIGURE 5:**
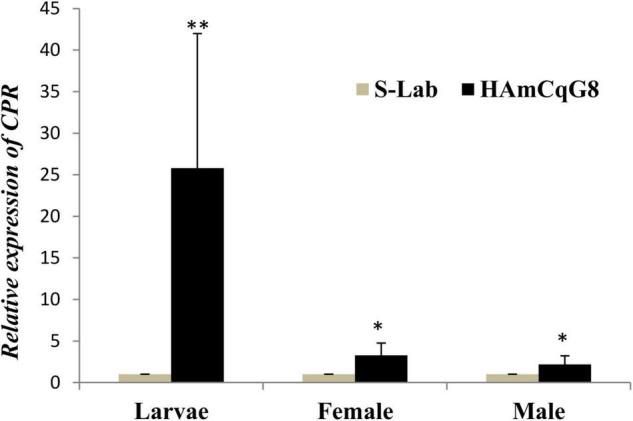
Cytochrome P450 reductase (CPR) relative gene expression in HAmCq^G8^ strain compared to the S-Lab strain. The relative levels of gene expression shown along the *Y*-axis represent the ratio of the gene expression in each sample of the resistant HAmCq^G8^ strain compared to that of the S-Lab strain. The results are shown as the mean ± S.E. A student’s *t*-test was used for the statistical significance analysis. Only both a value of *P* ≤ 0.05 and a cut-off value of a ≥2-fold change in expression (Strode et al., 2008) was considered as a statistically significant overexpression. *P ≤ 0.05, **P ≤ 0.01.

**FIGURE 6:**
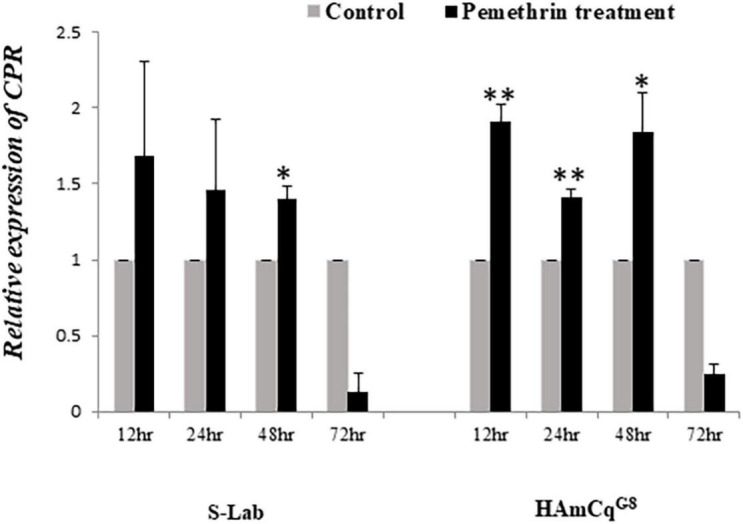
Induction expression of Cytochrome P450 reductase (CPR) in HAmCq^G8^ and S-Lab strains of *Cx. quinquefasciatus* following permethrin treatment. The relative expression of CPR gene in *Culex* mosquitoes following treatment with permethrin at their respective LC_50_ concentrations (0.005 and 10 ppm for the S-Lab and HAmCq^G8^ strains, respectively) were analyzed 12, 24, 48, and 72 h after the permethrin treatment. The relative levels of gene expression shown along the *Y*-axis represent the ratio of the gene expression in each treatment compared with that in acetone treated control mosquitoes (note that the *Y*-axis scales in the sub-figures varies). The experiments were repeated three times. The results are shown as the mean ± S.E. A student’s *t*-test was used for the statistical significance analysis. Only both a value of *P* ≤ 0.05 and a cut-off value of a ≥2-fold change in expression (Strode et al., 2008) was considered as a statistically significant induction expression. *P ≤ 0.05, **P ≤ 0.01.

The expression of CPR is up-regulated in both larvae and adults in the resistant HAmCq^G8^ strain compared to in the susceptible S-Lab strain. There is a 25-fold greater up-regulated in the larvae but just three and twofold increases in the female and male adults, respectively, in HAmCq^G8^ compared to S-Lab mosquitoes (Figure 5). These results indicate that CPR is uniquely highly over-expressed in the larval stage in resistant HAmCq^G8^ strain compared to in the S-Lab strain. No significant induction of CPR was found in either the S-Lab and HAmCq^G8^ strains at any time post- treatment (Figure 6), suggesting that the constitutive overexpression is contribute to the resistance in mosquitoes.

## Discussion

This study examined the co-expression of six P450 genes that are over-expressed in the permethrin resistant strain HAmCq^G8^ or MAmCq^G6^ strain with their co-factor cytochrome P450 reductase CPR in insect cells using a baculovirus expression system. Their role in the metabolism of permethrin and its intermediate metabolites PBOH and PBCHO was also investigated. In an earlier study of this functional mosquito P450-CPR co-expression system, we successfully co-expressed two proteins, CYP9M10 and CYP6AA7, and examined their role in the metabolism of permethrin (Gong et al., 2017). The current study builds on this earlier work, revealing that in addition to CYP9M10 and CY6AA7, six other P450 genes are also capable of metabolizing permethrin. This is not altogether surprising since researchers have predicted that these six P450 genes, which also belong to the CYP6 and CYP9 families, would be linked to insecticide resistance. Specifically, CYP9J40 was uniquely found to be transcriptional up-regulated in the larvae of HAMCq^G8^ strain and CYP9J33 was up-regulated in the larvae of the MAMCq^G6^ strain (another permethrin resistant strain) (Yang and Liu, 2011). CYP9J34, CYP6P14, CYP9J45, and CYP6BZ2 were all over-expressed in the larvae of both the MAMCq^G6^ and HAMCq^G8^ strains, suggesting a common mechanism for the development of permethrin resistance in these different resistant strains. Moreover, CYP9J33 can be induced by permethrin in resistant strain, MAMCq^G6^, while CYP9J34 and CYP9J45 were induced by permethrin in both the MAMCq^G6^ and HAMCq^G8^ strains (Gong et al., 2013). Among these six overexpressed P450 genes, CYP9J45, CYP6BZ2, and CYP6P14 have been demonstrated that decreased expression of these P450 genes corresponded with the decreased level of insecticide resistance to permethrin via RNAi analysis (Yang et al., 2021). Although some of these proteins have a relatively low turn-over rate for converting permethrin, their constitutive over-expression and induction expression profiles show that they can detoxify permethrin at a higher efficacy than is possible in susceptible strains. The results reported here thus provide further evidence of the central role played by multiple up-regulated P450 genes in the detoxification of insecticides in resistant mosquitoes. The over-expression of functional P450 genes in resistant insects is a common mechanism of insecticide resistance and multiple over-transcribed P450 genes belonging to the CYP6 and CYP9 families have been identified in resistant mosquito strains or populations (Müller et al., 2008; Strode et al., 2008; Yang and Liu, 2011; Reid et al., 2012; Stevenson et al., 2011, 2012; Riveron et al., 2013). Several have been validated by *in vitro* assays that show precisely how they metabolize insecticides (Müller et al., 2008; Stevenson et al., 2012; Chandor-Proust et al., 2013; Riveron et al., 2013). Up-regulation P450s with detoxifying functions have also been associated with insecticide resistance in other pest insects (Liu and Scott, 1998; Zhu et al., 2010; Nauen et al., 2013; Zimmer et al., 2014). For instance, it has been demonstrated that CYP6AY1 and CYP6ER1 metabolize imidacloprid and their over-expression in imidacloprid resistant *Nilaparvata lugens* has been implicated in the development of imidacloprid resistance (Bao et al., 2016; Pang et al., 2016). These reports clearly show that P450s play a key role in metabolic resistance in both agricultural and sanitary pests.

In a previous study, an expression mode for the co-expression of P450 and CPR was established using a baculovirus expression system (Gong et al., 2017). Building on this earlier research, the present study expressed six P450 proteins (CYP6P14, CYP6BZ2, CYP9J33, CYP9J34, CYP9J40, and CYP9J45) in a baculovirus expression system, identifying 100–450 pmol/mg P450 content and 43.6–162.5 nmol/min/mg CPR activity in isolated microsome proteins, confirming that the expression model was indeed suitable for the successful expression of P450/CPR functional complexes. The ECOD activity varied dramatically among these six expressed P450 proteins, ranging from 86.6 to 1682.4 pmol/min/mg. All but one of the proteins were capable of converting 7-ethoxy coumarin into 7-hydroxycoumarin, with the best ECOD activity for CYP9J33 and the lowest for CYP6P14. The differential efficiency of the substrate metabolism for individual P450s may be due to differences in their structures, leading to different substrate specificities: CYP6BQ23 prefers bulkier molecules such as 7-benzyloxymethoxy-4-trifluoromethyl coumarin (BOMFC), whereas 7-ethoxy coumarin was not significantly metabolized (Zimmer et al., 2014).

In the present study, the metabolic rates of permethrin mediated by these P450 proteins were similar to the values for CYP9M10 and CYP6AA7 observed previously (Gong et al., 2017), as well as to the results reported for *Anopheles gambiae* CYP6P3 (with a turnover of 0.97 min^–1^ for combined *trans* and *cis* permethrin; Müller et al., 2008) and for *Aedes aegypti* CYP9J32, CYP9J28, CYP9J26, and CYP9J24 (with a turnover of 0.8, 0.44, 0.6, and 0.16 min^–1^, respectively; Stevenson et al., 2012). However, no metabolite was detected in the HPLC analysis in the current study. This may be either because the amount of permethrin converted into PBOH was so small as to be undetectable, or the other metabolites present were not detected under the HPLC conditions used here. Somewhat surprisingly, none of the six proteins tested had any activity toward PBOH; all converted PBCHO to PBCOOH albeit with low efficiency. As shown in recent research, PBOH is the intermediate metabolite of permethrin, being produced either by the hydrolysis process of carboxylesterase (Gong et al., 2021) or from P450s through oxidative metabolic reactions (Nakamura et al., 2007; Gong et al., 2017). PBOH can be further oxidized into PBCHO or PBCOOH by P450s (Müller et al., 2008; Tangea et al., 2014; Gong et al., 2017), alcohol dehydrogenase or aldehyde dehydrogenase (Choi et al., 2002; Somwang et al., 2011). PBCOOH has a lower toxicity to *Culex* mosquitoes than either PBCHO or PBOH (Gong et al., 2017). Moreover, the permethrin resistant strain HAmCq^G8^ has a threefold higher resistance to PBOH and PBCHO than susceptible mosquitoes (Gong et al., 2017). These results all provide evidence that not only is the conversion of PBOH or PBCHO into PBCOOH beneficial for the survival of mosquitoes, but it also boosts their permethrin resistance by conferring PBOH and PBCHO tolerance. This means that the secondary metabolism of permethrin observed in our study would help more mosquitoes survive when exposed to permethrin and thus contribute to the development of resistance. The secondary metabolism of permethrin has also been reported in *A. gambiae*, and although two *A. aegypti* P450 genes, CYP6Z8 and CYP6Z2, cannot metabolize most insecticides they can metabolize PBOH and PBCHO, indicating that the secondary metabolism of insecticide pyrethroids by P450s is linked to resistance (Chandor-Proust et al., 2013).

The transfer of electrons from NADPH to the P450-substrate complex during P450 catalyzed metabolism is essential. It has been widely accepted that the P450 catalytic cycle involves the sequential transfer of two electrons from NADPH via P450 oxidoreductase, which is a CPR (Porter, 2012). Due to the crucial biological function of CPR in P450 systems, insect CPR is considered a vital part of P450-mediated insecticide resistance, with CPR being deemed a novel synergistic target (Liu and Scott, 1996; Lycett et al., 2006; Lian et al., 2011; Zhu et al., 2012). In the present study, we found that CPR is uniquely highly over-expressed in the larvae of permethrin resistant mosquitoes compared to the susceptible strain, indicating that its over-expression may be strongly linked to the over-expression of P450 genes. More P450 genes are found to be over-expressed in the larvae than adults in the permethrin resistant strain HAmCq^G8^, with 25 (11%) of the P450 genes overexpressed in the larvae and 17 (7%) in the adults (Yang and Liu, 2011). Moreover, in the larvae of HAmCq^G8^, 32% of the over-expressed P450 genes had a fivefold greater expression level than the susceptible reference strain, while only 12% of the over-expressed P450 genes in adult reached this level; in total, 68% of the genes that were over-expressed in resistant larvae had >3-fold expression level compared to the reference mosquitoes but only 41% in the adults did (Yang and Liu, 2011). These results indicate that far more P450s were over-expressed in the larvae of HAmCq^G8^ mosquitoes compared to the adults. It is therefore not surprising to find that CPR are highly over-expressed in the larvae of HAmCq^G8^ strains, at 25-fold but just 3- and 2-fold in females and males, respectively, compared to the S-Lab strain, or that CPR is co-over-expressed in permethrin resistant strains following permethrin selection. This co-over-expression enhances the P450-mediated detoxification of permethrin, ultimately contributing to the development of permethrin resistance. These results are consistent with our previous investigations, in which HAmCq^G8^ was highly resistant to pyrethroid insecticides after permethrin selections and exhibit a 2,700-fold level of resistance to permethrin at the fourth-larval stage compared to that in adult mosquitoes (100-fold), when they were all compared to the S-Lab strain, respectively (Xu et al., 2006; Li and Liu, 2010). The phenomenon of over-expression of CPR in resistant insects, along with P450 genes, has also been reported in other insects, including *Musca domestica* (Liu and Scott, 1996), *A. gambiae* (Nikou et al., 2003; Edi et al., 2014), and *A*. *Aegypti* (Marcombe et al., 2012), suggesting a possible role in resistance for the co-expression of CPR with functional P450s. The current study did not examine the precise function of CPR via RNAi analysis in *Culex* mosquitoes but its *in vivo* function has been linked to detoxification by P450s in other studies via RNAi analysis (Lycett et al., 2006; Liu et al., 2015), although these function studies were all conducted in insecticide susceptible populations. However, Zhu et al. (2012) examined the function of CPR in an insecticide resistant strain with over-expression of P450 genes by injecting dsRNA-CPR into bed bugs. They found the suppression of the *CPR* expression increased susceptibility to deltamethrin in resistant populations but not in susceptible bed bug populations. Their study strongly implied that the role of over-expressed CPR and P450s in detoxification is complex and P450-mediated metabolic detoxification may serve as one of several resistance mechanisms in bed bugs (Zhu et al., 2012). Tang et al. (2012) reported that silencing the P450 gene CYP6B7, either alone or together with CPR, increased the susceptibility of resistant *Helicoverpa armigera* to fenvalerate, suggesting that CYP6B7and CPR work collaboratively to enhance the metabolism of fenvalerate, thus playing an important role in the resistance of *H. armigera* to fenvalerate.

## Conclusion

Six P450 proteins, CYP6P14, CYP6BZ2, CYP9J33, CYP9J34, CYP9J40, and CYP9J45, were co-expressed in insect cells with CPR using a baculovirus expression system. *In vitro* enzymatic reactions and HPLC analysis demonstrated their important role in the metabolism of permethrin. Real time PCR analysis revealed that CPR is over-expressed in the larvae of permethrin resistant mosquito strains, suggesting the important role it play in the metabolism of permethrin and its involvement in the development of permethrin resistance. P450 –mediated metabolic resistance have been reported in many pest insects. This study examined the function of six over-expressed P450 genes in metabolizing permethrin in permethrin –resistant *Culex* mosquitoes. Although these six P450s did not metabolize PBOH, they were able to convert PBCHO to the less toxic product PBCOOH, indicating their likely involvement in the secondary metabolism of pyrethroids, together contributing to the development of permethrin resistance in *Culex* mosquitoes. This implies that secondary metabolism must also be considered in future research in this area. Significant up-regulation of CPR was also uniquely observed in the larvae of the permethrin resistant strain, with far more P450s being over-expressed and higher permethrin resistance than in either female or male adults. This strongly suggests that the co-expression of P450s with its co-factor CPR plays an important role in the permethrin detoxification process and hence the development of resistance. Taken together, these findings demonstrate the central role of multiple P450 genes and their co-factor CPR in the permethrin resistance of *C. quinquefasciatus*. These multiple P450 genes, along with their co-factor CPR, can thus be monitored to provide useful indicators of the incipient development of resistance where permethrin is used, or used as target genes for pyrethroid resistance management in mosquitoes.

## Data Availability Statement

The original contributions presented in the study are included in the article/supplementary material, further inquiries can be directed to the corresponding author.

## Author Contributions

NL, YG, TL, QL, and SL conceived and designed the study. YG and TL performed the experiments. NL, QL, and SL prepared the materials. YG and NL wrote the manuscript. All authors reviewed the manuscript.

## Conflict of Interest

The authors declare that the research was conducted in the absence of any commercial or financial relationships that could be construed as a potential conflict of interest.

## Publisher’s Note

All claims expressed in this article are solely those of the authors and do not necessarily represent those of their affiliated organizations, or those of the publisher, the editors and the reviewers. Any product that may be evaluated in this article, or claim that may be made by its manufacturer, is not guaranteed or endorsed by the publisher.

## Footnotes

1
https://vectorbase.org/vectorbase/app/record/dataset/DS_1b76ccf2da
